# Relationship of *SELE* A561C and G98T Variants With the Susceptibility to CAD

**DOI:** 10.1097/MD.0000000000001255

**Published:** 2016-03-03

**Authors:** Bihong Liao, Keqi Chen, Wei Xiong, Ruimian Chen, Aihuan Mai, Zhenglei Xu, Shaohong Dong

**Affiliations:** From the Department of Cardiology (BL, KC, WX, RC, AM, SD); and Department of Gastroenterology, Second Clinical Medical College of Jinan University, Shenzhen People's Hospital, Shenzhen, Guangdong, China (ZX).

## Abstract

Published genetic association studies have produced controversial results regarding the association of *SELE* gene polymorphisms (A516C and G98T) and CAD susceptibility. We therefore chose to perform a meta-analysis to determine the association.

Twenty-seven eligible articles were identified through electronic databases, providing 5170 CAD cases and 4996 controls. Fixed-effects or random-effects summary ORs were calculated to estimate the risk of CAD in relation to A516C and G98T. Forest plots and funnel plots were constructed by Stata software 12.0.

A strong association was observed between A516C and susceptibility of CAD among 4757 cases and 4272 controls. The summary OR was greatest in individuals carrying the CC genotype (OR = 1.91, 95% CI, 1.12–3.25). A significantly increased risk was indicated in both Caucasians and Asians. The analyses by disease type showed a significant increase in the risk of AP and MI. We also noted a strong association in population-based studies. In the analyses of G98T, data were available for 1422 cases and 1625 controls. We saw a markedly increased risk of CAD associated with G98T. The highest risk was indicated in individuals with the TT genotype (OR = 2.82, 95% CI, 1.15–6.89). A similar trend was seen in Asians and population-based studies.

These findings provide consistent evidence that A516C and G98T polymorphisms of the *SELE* gene may be associated with increased susceptibility of CAD.

## INTRODUCTION

Coronary artery disease (CAD) has become one of the major causes of death worldwide.^1^ CAD is a group of diseases including myocardial infarction (MI), angina pectoris (AP), and sudden coronary death. Despite the significantly declined global mortality, the occurrence rate remains especially high in developed countries.^2^ CAD is a multifactorial disease. Multiple traditional risk factors have been identified, but they can only explain a small fraction of reported cases.^3–10^ Thus, there must be other causes. Recently, much attention has been given to the hypothesis that CAD is a consequence of the inheritance of high- or low-penetrance genes involved in inflammation processes.^11,12^

The selectins are a class of cell adhesion molecules involved in chronic and acute inflammation processes. These single-chain transmembrane glycoproteins are expressed on endothelial cells after stimulation by inflammatory cytokines. The expression profile of selectins on the vascular wall is a determinant for the adhesion of pathological cells to endothelium, as the presence of endothelial selectins facilitates the adhesion. Accumulative data have implicated a linkage between selectins and the occurrence of CAD.^13–15^

E-selectin (SELE) is expressed on activated endothelial cells and plays a key role in the binding of circulating lymphocytes and monocytes to endothelial cells. The *SELE* gene is located at chromosome 1q22-q25 and contains 14 exons. Genetic polymorphisms at the *SELE* locus may upregulate the gene expression levels, and thus influence the biological functions of their protein.^16^ There is a A to C single nucleotide change at position 561 (A561C, rs5361). The common polymorphism lies in the epidermal growth factor-like domain of the *SELE* gene and results in arginine to serine (Ser128Arg) amino acid substitution at codon 128. Another polymorphism is a G to T nucleotide change in the 5’-untranslated region (G98T, rs1805193). Several lines of evidence have demonstrated significantly higher plasma SELE levels attributable to A561C and G98T polymorphisms.^54,55^ However, previous studies differ widely in their conclusions on the association between the *SELE* gene polymorphisms and CAD risk. In order to better define the association, we performed a meta-analysis incorporating 27 articles with 5170 CAD cases and 4996 controls.

## METHODS

### Search Strategy

Electronic databases of PubMed, ISI Web of Science, China Wanfang Database, and China National Knowledge Infrastructure (CNKI) were searched to seek all potentially relevant records written in English or Chinese language. We combined the following keywords:*SELE*, polymorphism and coronary artery disease, and their synonyms (E-selectin, rs5361, rs1805193, genetic variant, mutation, CAD; acute coronary syndromes, ACS; coronary heart disease,CHD; myocardial infarction, MI). Additional usable records were identified by scanning the reference lists of related original articles, narrative reviews, and meta-analyses. This study was approved by the ethic committee of Second Clinical Medical College of Jinan University, Shenzhen People's Hospital.

### Inclusion and Exclusion Criteria

Eligible studies were selected based on the predefined criteria. If several papers were conducted on the same CAD patients, the largest paper with complete data was considered eligible for inclusion.

Inclusion Criteria

1. Had a case-control/cohort design;
2. Evaluated the hypothesis that there was an association between *SELE* polymorphisms and CAD susceptibility;
3. Provided sufficient data to estimate odds ratios (ORs) with 95% confidence intervals (95% CIs);
4. Included human subjects.

Exclusion criteria

1. Published as an abstract or related forms of summary;
2. Reported too limited data to calculate the ORs;
3. Only patients were included;
4. Review articles and meta-analyses.

### Data Extraction

For the studies finally included in the meta-analysis, data on the following items were extracted by 2 independent reviewers: study design, total cases, and controls, first author, publication year, location of study, ethnicity, source of controls, disease type, diagnosis of cases, genotype numbers, and methods applied to determine the genotypes of A561C and G98T polymorphisms. Discrepancies were resolved through discussion including a third reviewer.

### Statistical Analysis

Statistical data were done using Stata software (v. 12.0; StataCorp LP, College Station, TX). Summary ORs were calculated to estimate the strength of association between CAD risk and *SELE* polymorphisms. Inter-study heterogeneity was detected with the *χ*^2^-based Q test, and *P* value less than 0.05 represented presence of heterogeneity. We calculated random-effects summary ORs using the DerSimonian and Laird method when the Q test indicated significant heterogeneity (*P* < 0.05); otherwise, we estimated fixed-effects summary ORs using the Mantel–Haenszel method. Hardy–Weinberg equilibrium deviation was evaluated by Fisher exact test among controls. Begg's funnel plot and Egger's linear regression test were performed to evaluate publication bias.^17,18^ Subgroup analyses were conducted by several characteristics (ethnicity, disease type, and source of controls) to assess the association for each subgroup. In addition, we performed sensitivity analyses to identify the studies that exerted significant influence on summary ORs.

## RESULTS

### Study Characteristics

**FIGURE 1:**
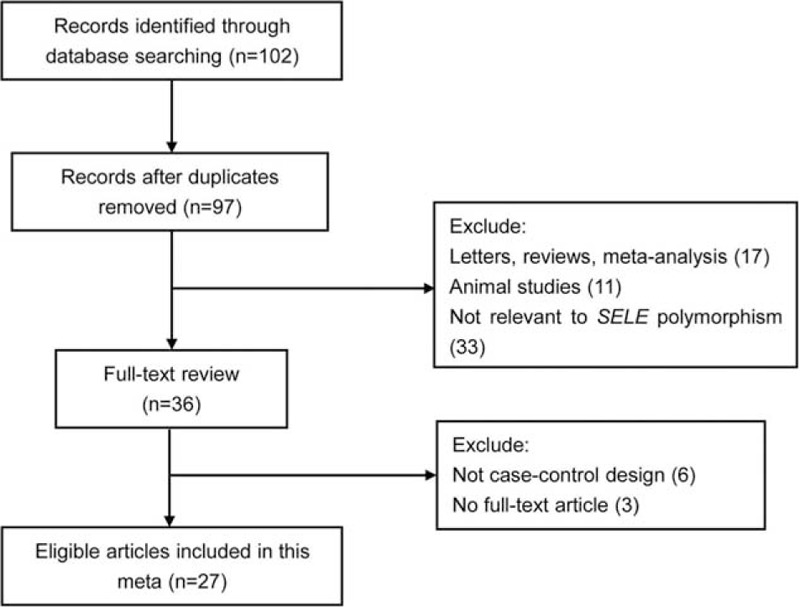
Flow diagram for literature selection.

The literature search according to inclusion and exclusion criteria yielded 102 records. Of these, 97 were retained after exclusion of duplicates. The review of title, abstract, and full text when necessary helped to exclude 70 more records. Thus, 27 articles on A561C and/or G98T polymorphisms and CAD were ultimately included in our analysis.^19–45^Figure 1 shows the detailed process of study selection. Information on the principal characteristics of each included study is presented in Table 1 .

**TABLE 1:**
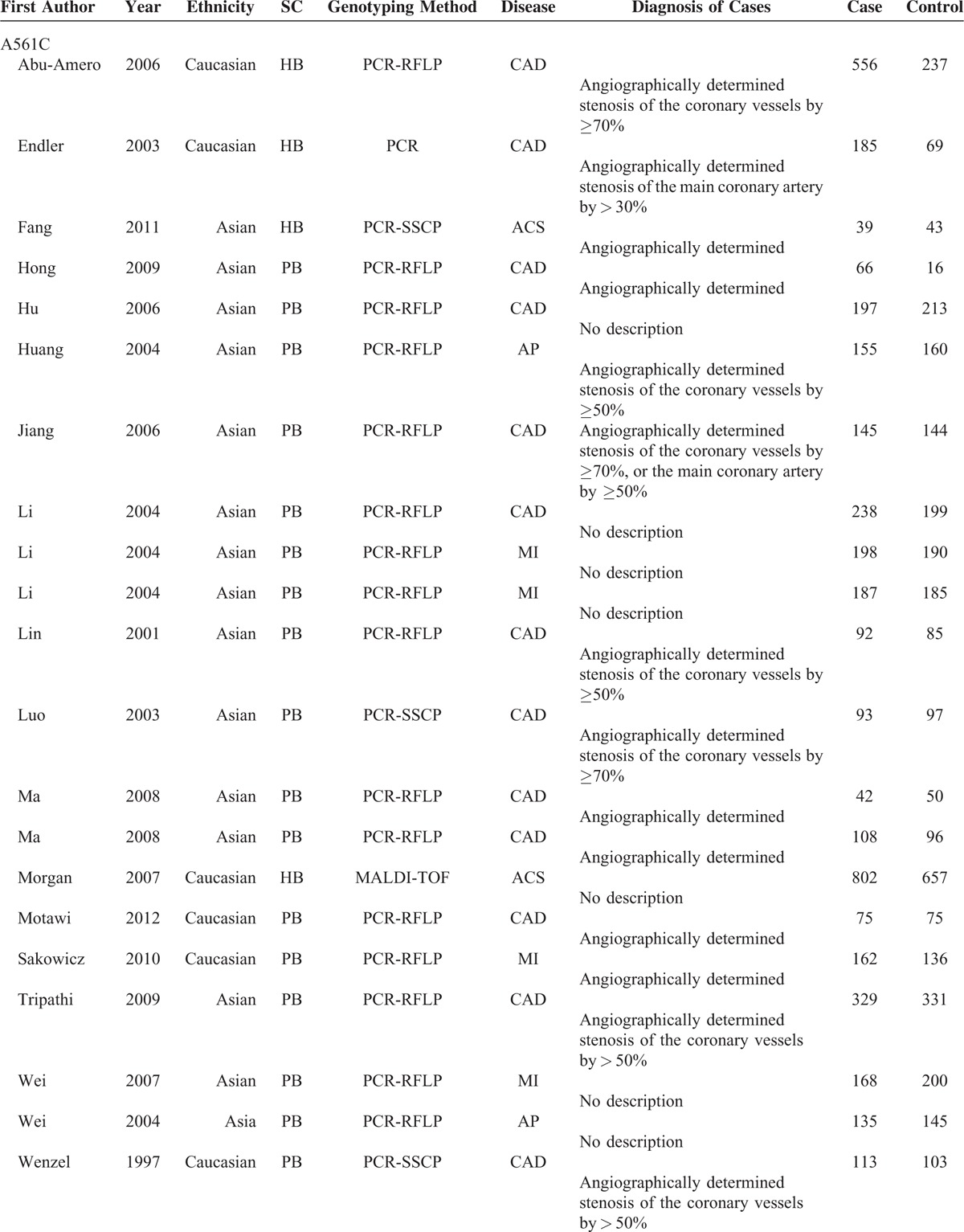
Principal Characteristics of the Studies Included in the Meta-Analysis

**TABLE 1 (Continued):**
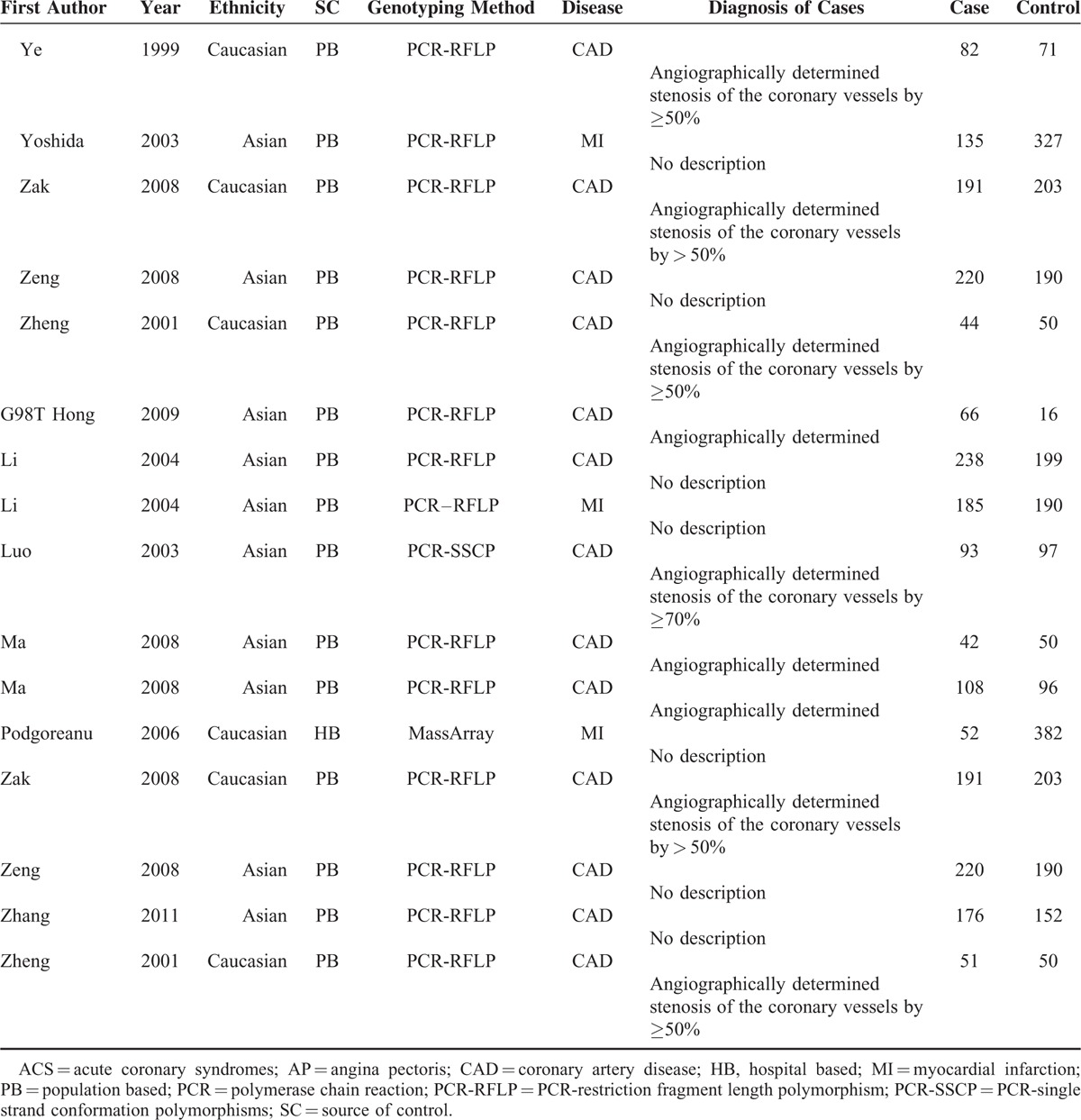
Principal Characteristics of the Studies Included in the Meta-Analysis

### Meta-Analysis Results

**FIGURE 2:**
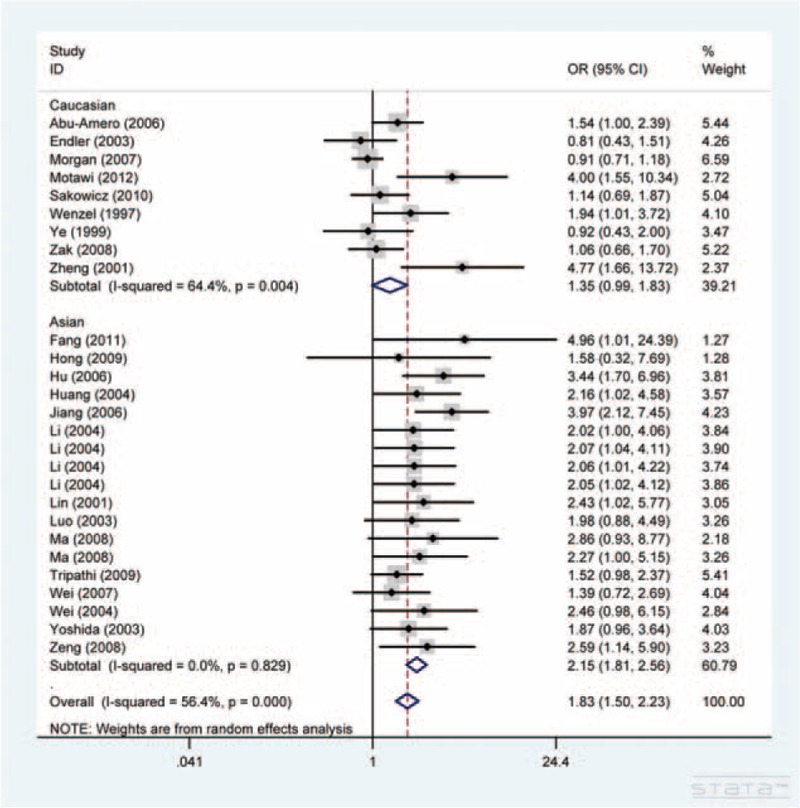
Forest plot for the association between *SELE* A561C polymorphism and CAD susceptibility in CC/CA versus AA model.

In the meta-analysis of A561C polymorphism and CAD risk, data were available for 4757 cases and 4272 controls included in 24 articles. As shown in Table 2, the A561C polymorphism was strongly associated with the risk of CAD at both the allelic and the genotypic level. The strongest association was shown in individuals carrying the CC genotype (OR = 1.91, 95% CI, 1.12–3.25, CC vs. AA). We proceeded to estimate the risk using CC/CA versus AA model, because 8 studies (see notes below Table 2) with 1485 cases and 1603 controls were excluded in the previous analysis assuming CC versus AA model because of the count of CC genotype in both cases and controls was 0. A similar significant increased risk was indicated in individuals with CC/CA genotypes (OR = 1.83, 95% CI, 1.50–2.23, Figure 2).

**TABLE 2:**
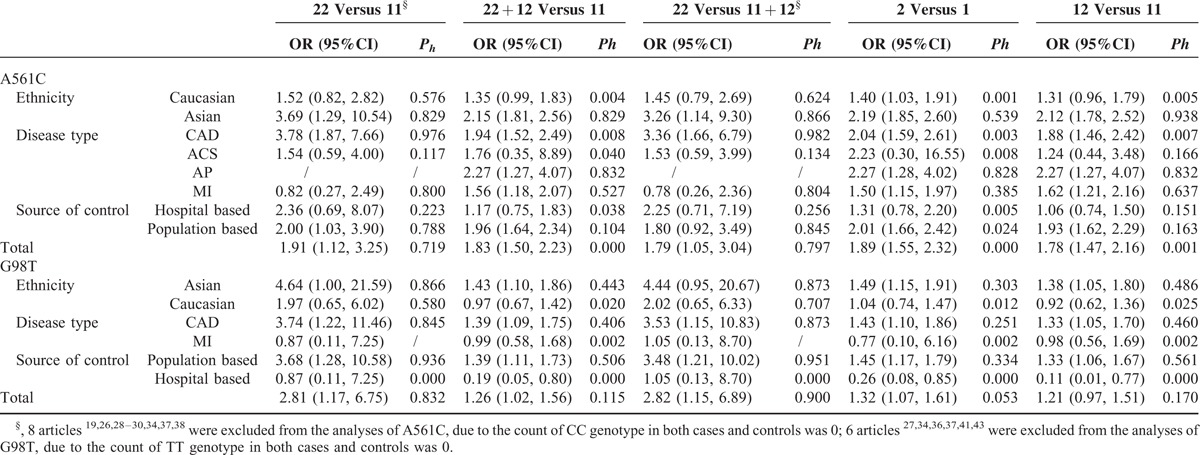
*SELE* Polymorphisms and CAD Risk

In the subgroup analyses by ethnicity, we noted a strong association for Caucasians and Asians (Figure 2). Final analyses by disease type and source of controls indicated significantly increased risk for angina pectoris (AP), myocardial infarction (MI), and population-based studies (Table 2).

**FIGURE 3:**
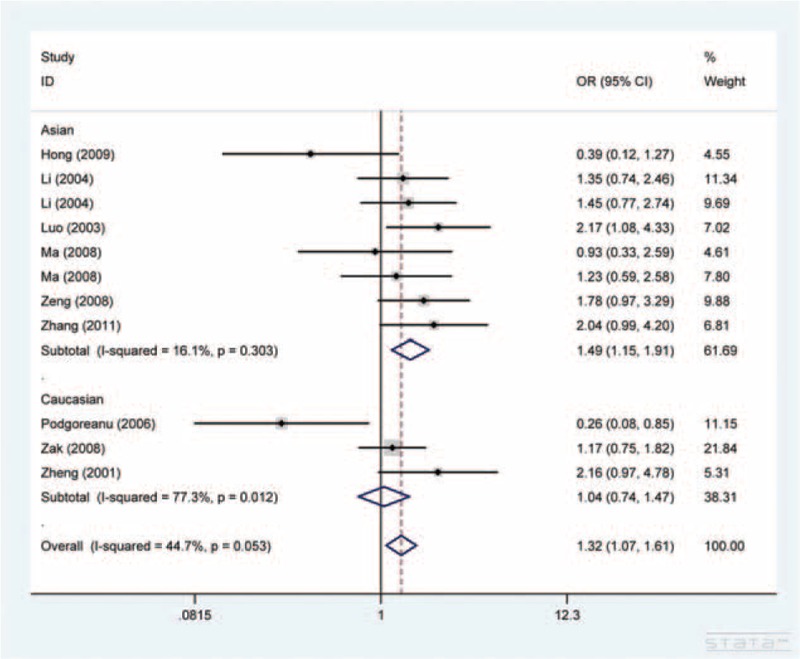
Forest plot for the association between *SELE* G98T polymorphism and CAD susceptibility in T versus G model.

Meta-analysis of G98T polymorphism and CAD risk was based on 1422 cases and 1625 controls combined from 10 articles. We observed a strong association in all models tested with the exception of GT versus GG. The OR was greatest in TT versus GT/GG model (OR = 2.82, 95% CI, 1.15–6.89) and relatively lower in T versus G model (OR = 1.32, 95% CI, 1.07–1.61, Figure 3). Further analyses indicated a strong association for Asians (Figure 3), population-based, and hospital-based studies (Table 2).

### Heterogeneity Test and Sensitivity Analysis

Several analyses of A561C polymorphism demonstrated presence of heterogeneity between studies (*P* < 0.05). In order to identify the outliers, we performed sensitivity analyses. The results were homogeneous when 2 studies, published by Jiang et al and Morgan et al^32,44^, respectively, were removed (*P* > 0.05). Although no significant heterogeneity was indicated in the analyses of G98T polymorphism, we also conducted sensitivity analyses to examine the stability of each risk estimate. The results suggested no substantial influence on the pooled ORs (data not shown).

### Publication Bias

**FIGURE 4:**
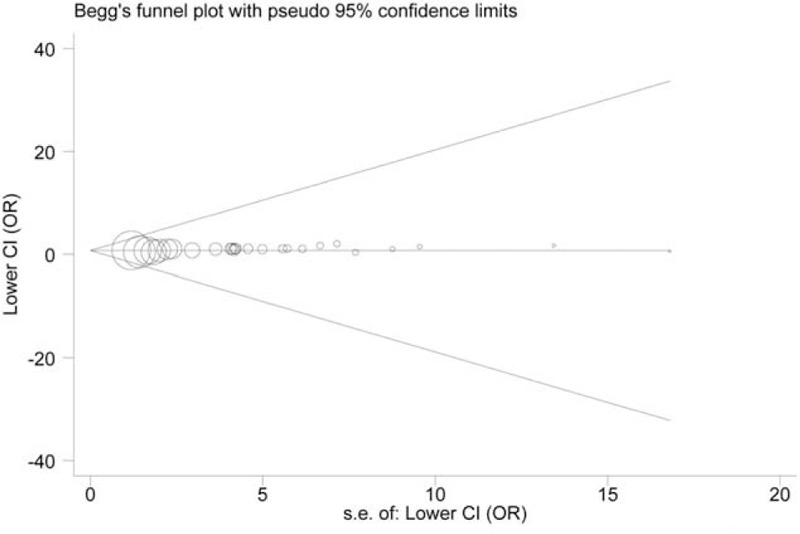
Begg's funnel plot of the A561C polymorphism and CAD risk.

**FIGURE 5:**
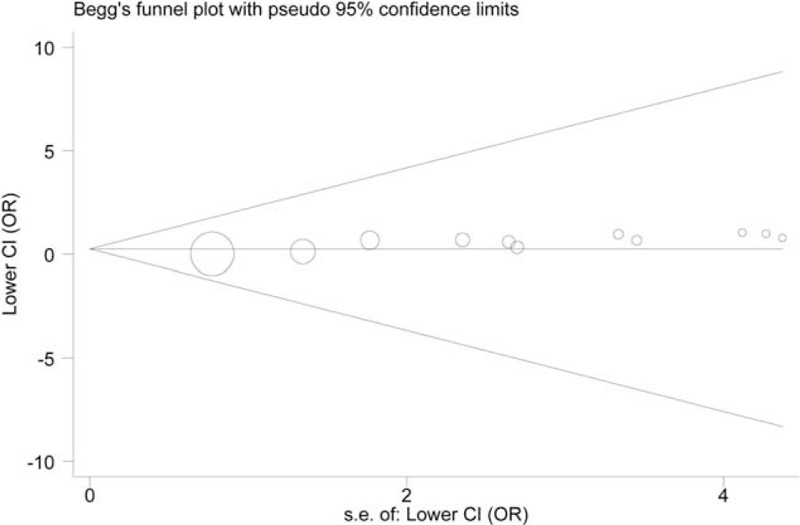
Begg's funnel plot of the G98T polymorphism and CAD risk.

The circles, corresponding to the single studies, were symmetrically distributed in the funnel plots created for A561C and G98T polymorphisms (*P* > 0.05). However, Egger's test suggested evidence of significant publication bias in CA versus AA model of A561C polymorphism, and in GT versus TT model of G98T polymorphism (*P* < 0.05). The funnel plots are shown in Figures 4 and 5, respectively.

## DISCUSSION

CAD is considered to be the number 1 cause of death in both males and females in many parts of the world.^46^ While previous research has established a broad range of risk factors, no factor has consistently been linked to CAD risk except for the formation of coronary atherosclerosis.^47,48^ Mounting evidence has suggested inflammation as an important component in the pathological process of coronary atherosclerosis.^49,50^ The *SELE* gene is a known cell adhesion molecule with a significant role in inflammation. There is much speculation of a possible linkage between *SELE* gene polymorphsisms and the development of CAD. Many groups have previously evaluated the hypothesis in a variety of populations.^40,51,52^ Nonetheless, the reported associations are conflicting. The discrepancies may be as a result of ethnically diverse populations and different sample sizes. Other factors, such as different study design may also result in the controversial results. These common limitations of single studies can be overcome when meta-analysis is used.

In our meta-analysis, genotype data were available for a total of 5170 CAD cases and 4996 controls pooled from 27 published papers. We provided some evidence that seemed to support a strong association between *SELE* gene polymorphsisms and CAD risk. Presence of the A561C polymorphism was associated with a 1.78 to 1.91 fold higher risk of CAD. The G98T polymorphism showed a stronger association with the disease development (summary ORs ranged from 1.26–2.82). These findings are biologically plausible, as single nucleotide polymorphisms at the *SELE* locus affect normal functions of the gene by upregulating the gene expression levels.^53^ The observation of upregulated expression levels of the *SELE* gene due to polymorphisms is supported by Bannan et al, who found significantly higher levels associated with the C allele at the A561C polymorphism.^54^ A more recent study by Mlekusch et al lend further support for the significant association between increased plasma SELE levels and the *SELE* gene polymorphisms.^55^

We identified 3 published meta-analyses evaluating the risk of CAD in relation to the A561C polymorphism and/or the G98T polymorphism. In the first meta-analysis, Wang et al identified 24 articles, 3 articles less than our analysis, showing a significant association of the 2 *SELE* gene polymorphisms with CAD risk.^56^ However, they did not analyze the risk of several disease types, such as AP and MI. In our study, we found a strong association between A561C polymorphism and the risk of AP and MI. The second meta-analysis was published by Dong et al.^57^ A total of 3193 CAD cases and 3233 control subjects were pooled from 20 case-control studies. Unlike the first meta-analysis, Dong et al did not demonstrate a positive association for Caucasians and MI. These observations were not consistent with the present study where more than 3500 subjects (1977 cases and 1763 controls) were included compared with the second analysis. In the most recent analysis, Wu et al only identified 10 studies with 3369 cases and 2577 controls for the A561C polymorphism,^15^ and found an increased risk for Asians, but not for Caucasians. Unlike the 2 more recent analyses, our analysis in which 1464 more subjects of Caucasian origin were combined even compared with the larger study, indicated a significantly higher risk of CAD associated with the C allele at A561C polymorphism. Therefore, the failure to include all informative studies may have biased some conclusions of the previous meta-analyses.

Certain limitations need to be noted in explanation of the present findings. First, although we have identified all published papers, they total sample is still relatively small and remains to be expanded to validate the positive associations suggested in the current study. Second, Egger's test showed evidence of significant publication bias that may be caused by the inclusion of several small studies. In addition, there was large inter-study heterogeneity in the results of some analyses. Possible causes include heterogeneous experimental designs, different subject populations, interventions, and choice of analysis. Third, CAD is a heterogeneous disease. Both environmental and genetic factors could contribute to the malignant development. But additional analyses to detect the effects of gene-environment interactions were not allowed because of lack of related data.

In conclusion, our data suggested a significant association of the A561C and G98T polymorphisms at the *SELE* locus with higher risk of CAD. This study may help to better understand the biological mechanism underlying the pathology of CAD, and this might ultimately facilitate earlier diagnosis, improved prevention and treatment. The role of *SELE* gene polymorphisms in the etiology of CAD remains to be elucidated in a larger study.
